# Convex Grooves in Staggered Herringbone Mixer Improve Mixing Efficiency of Laminar Flow in Microchannel

**DOI:** 10.1371/journal.pone.0166068

**Published:** 2016-11-04

**Authors:** Tae Joon Kwak, Young Gyu Nam, Maria Alejandra Najera, Sang Woo Lee, J. Rudi Strickler, Woo-Jin Chang

**Affiliations:** 1 Mechanical Engineering Department, University of Wisconsin-Milwaukee, Milwaukee, WI, United States of America; 2 Industrial Engineering Department, University of Wisconsin-Milwaukee, Milwaukee, WI, United States of America; 3 Department of Biomedical Engineering, Yonsei University, Wonju, Republic of Korea; 4 Great Lakes Water Institute, University of Wisconsin-Milwaukee, Milwaukee, WI, United States of America; Technion Israel Institute of Technology, ISRAEL

## Abstract

The liquid streams in a microchannel are hardly mixed to form laminar flow, and the mixing issue is well described by a low Reynolds number scheme. The staggered herringbone mixer (SHM) using repeated patterns of grooves in the microchannel have been proved to be an efficient passive micro-mixer. However, only a negative pattern of the staggered herringbone mixer has been used so far after it was first suggested, to the best of our knowledge. In this study, the mixing efficiencies from negative and positive staggered herringbone mixer patterns as well as from opposite flow directions were tested to investigate the effect of the micro-structure geometry on the surrounding laminar flow. The positive herringbone pattern showed better mixing efficiency than the conventionally used negative pattern. Also, generally used forward flow gives better mixing efficiency than reverse flow. The mixing was completed after two cycles of staggered herringbone mixer with both forward and reverse flow in a positive pattern. The traditional negative pattern showed complete mixing after four and five cycles in forward and reverse flow direction, respectively. The mixing effect in all geometries was numerically simulated, and the results confirmed more efficient mixing in the positive pattern than the negative. The results can further enable the design of a more efficient microfluidic mixer, as well as in depth understanding of the phenomena of positive and negative patterns existing in nature with regards to the surrounding fluids.

## Introduction

Microfluidic devices are defined as devices in which at least one of the dimensions is less than a millimeter. Major applications of microfluidic devices are manipulation and detection of chemicals and biological molecules such as DNA, proteins, and cells, related to food, health,[1] drug screenings, etc.[2] In this micro-scale analytical device, mixing is one of the most important properties to improve its performance, due to the creation of laminar flow, which has high momentum diffusion and low momentum convection. The laminar flow is explained by a low Reynolds number that is conventionally less than one in a microfluidic device [3]. Thus, mixing is difficult to achieve in a microfluidic channel; therefore, various micromixers have been developed [4,5].

The micromixers are frequently classified into active and passive mixers. Active mixers rely on external sources that can physically agitate the liquid in a microchannel, such as acoustic or ultrasonic waves with acoustic actuators [6], magnetic disturbances with magnetic particles and an external magnetic field [7], pressure disturbance with repeated stopping and flowing of the fluids, etc. [8] On the other hand, passive mixers do not use an external force, but mostly rely on hydrodynamic manipulation of the fluids, for example, chaotic advection [9,10], enhanced molecular diffusion [11], utilization of surface tension [12], fluid lamination [13], and sequential splitting and combining of fluids inside the microchannel [14]. Due to its simple design and fabrication, as well as higher mixing efficiency, chaotic advection is one of the most commonly used passive mixers. Chaotic micromixers consist of geometries embedded into the microchannels to disturb laminar flow.

One of the most efficient chaotic micromixer is the Staggered Herringbone Mixer (SHM) developed by Stroock et al [10]. The SHM consists of repeated patterns of grooves on the bottom of the microchannel. The individual groove is composed of two different length channels connected to each other, one relatively longer and the other relatively shorter, and those two grooves meet with a certain angle, usually 45 degrees. The grooves create helical motions of the moving fluid inside the microchannel. Ansari and Kim [15] focused on the SHM mixing performance, simplification, and geometry optimization regarding the depth and angle of the groove, and they found that mixing is more affected by the depth of the groove than the angle. The higher mixing efficiency with deeper grooves SHM was supported by numerical simulations as well. Several groups have numerically [10,16,17] and experimentally [18,19] investigated mixing efficiency and developed analytical models to characterize mixing in the SHM. The SHM shows relatively higher mixing efficiency when the Reynolds number is in a range of 0 < N_Re_ < 100, and the mixing effect remains qualitatively the same when N_Re_ is larger than 100 [16]. Also, two counter-rotating grooves composed of two mirrored shapes of long- and short-grooves in the SHM generate the rotation motions of the spiral flow to transport mass; therefore, the set of grooves enhance the mixing [20].

However, all of the SHMs investigated to date use negative grooves that have negative concave structures below the bottom surface of the microchannel, to the best of our knowledge. It is assumed that the effect of positive patterns has not been investigated, because the geometry of the herringbone area is the same for both of negative and positive patterns. The major difference between positive and negative patterns is convex or concave geometry at the beginning of each herringbone pattern.

In this study, we fabricated a new SHM that has positive convex grooves pattern and compared the mixing efficiencies with conventional negative patterns. The positive grooves pattern is convex structures above the bottom surface of the microchannel. Also, the effect of flow direction was investigated using an inverted groove shape for the first time. The mixing efficiencies were compared by using the number of SHM cycles required to complete mixing superficially. Also, experimental results were compared with numerical simulation, and the behaviour of the fluids around different SHM structures was simulated.

## Materials and Methods

### Experimental setup and sample preparation

The two liquids, fluorescein (Catalog number 46955, 0.001% w/v, Sigma-Aldrich Co., USA) and DI water were filled in two 100 μL glass syringes (Hamilton Co., USA). Each syringe was connected to one of two inlets of the SHM device through 30 cm long PTFE tubing (0.016” OD, 0.004” ID, EW-06417-72, Cole-Parmer Co., USA). Two syringe pumps (KDS-250, KD Scientific, USA) were used to inject the two fluids with 0.36 μL/min of flow rate each. The SHM device was made of poly dimethyl siloxane (PDMS, Sylgard 184, Dow-Corning Co., USA) by a casting method using a mold fabricated by photo-lithography processes. For the viscous fluids experiments, viscosities of the solutions were adjusted by changing the concentration of glycerol (G6279-500ML, Sigma-Aldrich Co., USA) solution in DI water.

### Microfluidic devices design and fabrication

**Fig 1:**
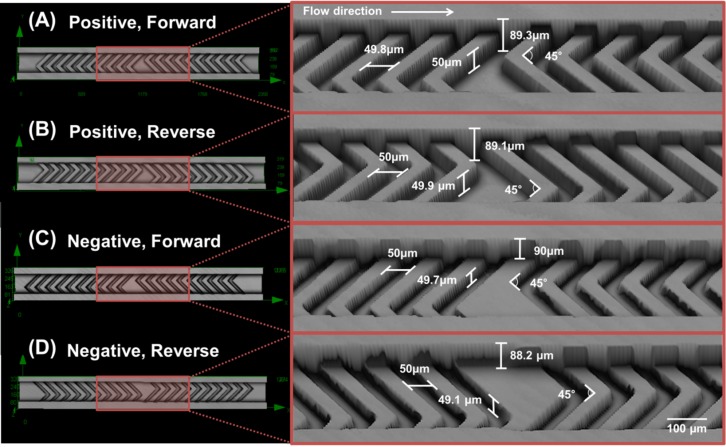
3D images of the SHM devices used in the experiments. (A) Positive forward (B) Positive reverse (C) Negative forward (D) Negative reverse. Size of scale bar is 100 μm. Detailed dimension of the structure is displayed in magnified pictures on the right of each image.

Fig 1 shows a picture of 1 cycle in the SHM using a 3D confocal laser scanning microscope (LEXT OLS 4100, Olympus Co., Japan). One cycle of the SHM is composed of 10 short-and-long grooves and another 10 long-and-short grooves as shown in the pictures on the left of Fig 1. The fluids entered from left to right. A total of 10 cycles are fabricated in a device. The spacing between two cycles is 500 μm.

The effect of the flow direction on mixing efficiency has never been surveyed so far. Thus, an SHM with an inverted shape was fabricated in addition to the normal shape for both positive and negative grooves. The normal SHM shape is indicated as “Forward” in Fig 1A and 1C, while the inverted shape is indicated as “Reverse” in Fig 1B and 1D. Both forward and reverse devices were fabricated in the same mold to avoid height variations originating from separate photolithographic fabrications. Thus, two molds, i.e. one for positive and the other for negative SHM, were fabricated.

The height of the microchannel that has the SHM structure is designed to 90 μm. The width of the microchannel is 200 μm. The width and angle between long and short grooves is 50 μm and 45°, respectively. The groove depth is a critical factor to affect the mixing efficiency. Aubin et al. [20] demonstrated the grooves that are 30% deeper than the channel height have a higher mixing efficiency, given that it promotes spatial homogenization without increasing the pressure in the mixer. Hence, both negative and positive groove depths were fabricated at a height of around half of the main channel height. Detailed measured dimensions are included in the pictures on the right of in Fig 1. The positive pattern has a concave SHM structure on the bottom of the microchannel. Thus, the distance between the top surface of the SHM structure and the cover PDMS is around 40 μm, since the height of the SHM structure is around 50 μm, as shown in Fig 1A and 1C. On the other hand, the negative pattern has a convex SHM structure on the bottom of the microchannel, therefore the distance between the bottom of the SHM structure and the cover PDMS is around 140 μm as shown in Fig 1B and 1D. The device-to-device variation in height of the microchannels fabricated on the same mold is less than 2 μm. A device with no SHM was fabricated to compare the diffusive mixing effect (pictures not shown). The height of the microchannel with no SHM is 90 μm as well. The picture of the device used in this experiment is shown in the supplementary material (S1 Fig).

Two-step lithography is required for fabrication, since both positive and negative grooves pattern of the SHMs have different heights in a device. First, a layer of negative photoresist (SU-8 50, MicroChem Co., USA) was spin coated on a silicon wafer and then soft-baked at 65°C for 5 minutes and then 95°C for 10 minutes. After exposure to UV light through a photomask, post-exposure-baking was done at 65°C for 1 minute and then 95°C for 3 minutes. Then, the second layer of the same SU-8 was spin-coated. The second photomask was carefully aligned and exposed to the UV light. A mask aligner (PLA - 501F, Canon Co., Japan) was used for photolithography. The mold was developed after post-exposure-bake to finally obtain the master mold for PDMS casting. The surface of the master mold was functionalized with trichloro (3, 3, 3-trifluoropropyl) silane (Sigma-Aldrich, USA) for easily stripping the hardened PDMS after molding. PDMS polymer and cross linker was mixed with the ratio of 10 to 1, and poured to the mold. The mold was left in an oven for approximately an hour at 85°C to harden the PDMS, which was then removed from the mold. It was bonded to a flat PDMS layer after Oxygen plasma treatment (ZEPTO, Diener electronic Co., Germany), following hole punching to inject fluids. Thus, all the surfaces inside the microchannel are PDMS.

### Measurement of mixing

The fluorescence pictures were taken using a cooled CCD camera (DP-72, Olympus, Japan) and fluorescence microscope (BX53F, Olympus, Japan) with a fluorescent light source (Lumen Dynamics X-Cite Series 120 Q, Excelitas Technologies Co., USA) and Microscope Light Power Supply (TH4-100, Olympus, Japan). The 20x objective lens was used, and the exposure time and ISO of the camera was set to 200 ms and 200 to take images, respectively. The resolution of the obtained image is 1360 by 1024 pixels (2/3 CCD, 6.45 μm pixels pitch). The scanned intensity of fluorescence across the microchannel was determined by averaging the brightness of 650 pixels (across the channel) by 200 pixels (along the channel) area right after each SHM cycle; the size of area in the image is equivalent to 214.5 μm (across the channel) by 62 μm (along the channel) Consequently, the fluorescence intensity scanned across the microchannel is an average of the brightness of 200 pixels using ImageJ image analysis software (National Institutes of Health, USA). The number of cycles required to complete mixing was determined by comparing the scanned brightness data across the microchannel. The number of cycles required for complete mixing was decided by comparing the scanned brightness profile with the profile after the previous cycle using the ANOVA test with a 95% confidence level.

### Numerical Simulation

The numerical simulation was performed to solve incompressible Newtonian fluid by Navier-Stokes equations and convection-diffusion equations [21] for each SHM devices using the finite element solver (COMSOL Multiphysics v5.0, Comsol Inc., Sweden)[22,23]. The equations are solved for a steady state flow, and each of the inlet flows was set as laminar flow, the pressure of the outlet channel was set as 0 Pa, velocity of the flows at the channel walls was set as 0 m/s (no slip at all the solid surfaces), and the fluorescence dye concentrations at two inlets was set as 1 (upper inlet) and 0 (lower inlet). The equations were solved using the generalized minimal residual method (GMRES) solver for a mesh with an elements number of 6.96–7.10 × 10^6^ and average element sizes of 1.7–2.0 μm depending on SHM pattern geometries. To verify the efficiency of each shape of the groove patterns, the cross-sectional images after each cycle were obtained. All the dimensions of the SHM and microchannel used in the numerical simulation were identical to the experiments. Also, the height of the microchannel and SHM structure were changed to simulate the behavior of fluids around the SHM structure that have different heights.

In addition to the comparison of the channels used in the experiments, the overall height of the negative pattern channels with groove depth was lowered to equalize the overall height of positive pattern channels. The groove depth of the shallow negative pattern channel was 50 μm, and the width and height of the microchannel was 200 μm, and 40 μm, respectively without grooves. Moreover, the mixing efficiency of shallow negative patterns was simulated to compare with the mixing efficiency of the positive patterns.

## Result and Discussion

### The diffusion of fluorescein in microchannel without SHM

Molecular diffusion is the major driving force of mixing in a microchannel due to laminar flow, if no mixer is integrated. The characteristics of diffusion in a microchannel are well defined experimentally and numerically. Kamholz et al. [24,25] studied a theoretical analysis of the scaling laws of diffusion in a microfluidic chemical analysis device with pressure-driven flow by correlating the non-uniform velocity profile and induced distribution of analyte molecules with residence time. In the scaling laws, the widening of diffusion area near the top and bottom walls in the channel is larger than that at the half depth of the channel. They adopted a custom two-dimensional model that describes convection and diffusion in a system of two fluids. The non-uniform diffusion was identified as the butterfly effect, and the scaling law has been reported across the channel depth by a two-dimensional finite difference simulation depending on the channel aspect ratio (width to depth). Lee et al. [26] surveyed the optimum flow rates for bioluminescent reaction in a microfluidic device when no mixer was integrated. Adenosine triphosphate (ATP) was used as a substrate of light producing enzyme, luciferase. It is recommended to inject the relatively lower molecular weight ATP solution in the middle with the two higher molecular weight luciferase solutions on both sides for higher diffusive mixing in a long microchannel. An almost negligible flow rate close to the surface of the channel due to the non-slip condition, and relatively faster flow rate in the center area enhance this effect.

**Fig 2:**
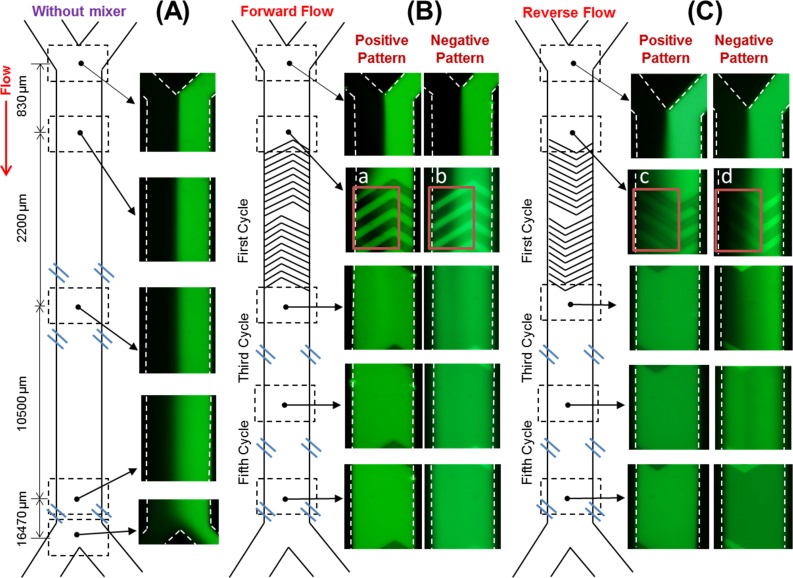
Mixing of DI water and fluorescein solution. In blank (A), and SHM device with forward (B) and reverse (C) flow.

The diffusion of fluorescein in the blank microchannel without a mixer is shown in Fig 2A. The length and total volume of the main channel except inlet and outlet branches were 30 mm and 0.51 μL, respectively. The retention time of the fluids was 0.7 min, because total flow rate of fluorescein and DI was 0.72 μL/min. The Reynolds number is calculated from ρdv/μ where ρ is density of the fluid, d is the hydraulic diameter of the microchannel, v is the velocity of the fluid, and μ is the viscosity of the fluid. In this case, density and viscosity of the water were used to calculate the Reynolds number, because the concentration of the fluorescein was very low (0.001%). The calculated Reynolds number is 0.11 for the blank device, and thus the flow is laminar. The diffused length of the fluorescein before exit was 26.1 μm, and the calculated diffusivity of the fluorescein in water is 0.80 nm^2^/s. This is in a comparable range with the diffusivity calculated by Fick's second law [27],[28] to determine the diffused distance of the fluorescein when the diffusivity in water is 0.64 nm^2^/s [29].

### Mixing with SHM

The pictures of the SHM structures are shown in Fig 1 in the Methods section. Using these channels, the fluorescence images of all four operation modes of SHM, i.e. positive forward, positive reverse, negative forward, and negative reverse, are shown in Fig 2B and 2C. The SHM is composed of a number of repeating patterns, i.e. cycles, and each repeating pattern has 10 short-to-long grooves and another 10 long-to-short grooves, as described earlier. All devices have a total of 10 cycles in this experiment. The mixing of the two fluids was completed before exit (after 10 cycles) in all four devices. The total volume of mixing area for the positive pattern device is 0.47 μL and for the negative pattern device is 0.61 μL. The total volumes are different, because both devices were designed to have the same height in inlet and outlet branch area. The positive convex pattern and negative concave pattern from the branch area reduces and increases the total volume, respectively. The total volume is calculated based on the measured dimension of the microchannel. The calculated Reynolds numbers for the positive and negative pattern devices are 0.229, and 0.097, respectively, because total flow rate of fluorescein and DI was 0.72 μL/min. The Reynolds number is below 1 in all devices, and thus the flow is laminar. Also, the negative SHM device shows slightly lower Reynolds number due to larger internal volume than the positive device with the same total flow rate. However, the effect of flow on mixing in this low Reynolds number range is insignificant.

The behavior of the fluorescence at the beginning of first SHM cycle (highlighted in red rectangles in Fig 2B and 2C) shows different effects depending on the groove geometries and flow directions. As shown in the red rectangle on the left side of Fig 2B and Fig 2B-a, the fluorescein solution in positive forward flow follows the shape of the groove as it is entering the first cycle, unlike the negative forward flow where the fluorescein solution only fills the groove as shown in the red rectangle on the right side of Fig 2B and Fig 2B-b. Thus, the fluorescein solution reached to the opposite side wall after the first positive groove (top left side of the Fig 2B-a) as the inter-space between the first and second groove is filled with fluorescein up to left-side wall. On the contrary, the inter-space between the first and second grooves is not completely filled with fluorescein with negative grooves (bottom left side of the Fig 2B-b). Thus, positive SHM is expected to have a better mixing effect compared with conventionally used negative grooves. When the fluid flows reverse direction, the fluorescing area in the negative SHM (Fig 2C-d) is smaller than that of the positive (Fig 2C-c), because the groove guides the fluid to the fluorescein side (right side of the pictures) in both the positive and negative patterns. However, it can be seen that the fluorescence solution flowed more towards the left side in the positive pattern (Fig 2C-c) than the negative pattern (Fig 2C-d). Obviously, the fluorescein could not reach the opposite side wall in both positive and negative patterns with reverse flow (in the Fig 2C-c and 2C-d). Based on the observations of the width of the fluorescein transferred across the microchannel after a few grooves from the entrance of the SHM cycle, it is confirmed that the positive pattern affects the mixing of the fluid more than the negative. As already known, repeating alternated patterns (short-to-long and long-to-short) also enhance the mixing significantly. The fluorescence images after the first, third, and fifth cycles are also shown in the third, fourth, and fifth row in Fig 2B and 2C, respectively. The mixing was incomplete in all four operation modes after the first cycle. Based on the pictures after the first cycle, the mixing effect was lowest in reverse negative SHM. The mixing was almost completed after the fifth cycles, and completed in all four operation modes after 10 cycles (data not shown).

**Fig 3:**
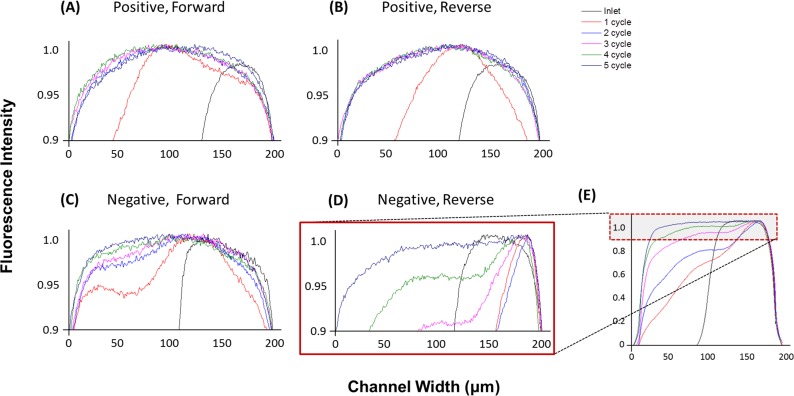
Normalized fluorescence intensity across the microchannel after each cycle from 1 to 5. (A) Positive forward (B) Positive reverse (C) Negative forward (D) Negative reverse (E) Negative reverse with full scale of fluorescence intensity.

The intensity of the fluorescence across the microchannel was scanned after each cycle from 1 to 5 and the expanded view from 0.9 to 1.0 of normalized intensity is displayed in Fig 3. The intensity of the fluorescence at the branch area varies slightly even with the same setup and fluorescein solution. Also, the highest intensity is decreasing with the progress of the mixing. Thus, the obtained intensity from the scanning of the captured fluorescence images were normalized in each analysis by dividing all the scanned brightness data by the maximum brightness of the fluorescence in each set of data. Thus, the location shows maximum brightness is normalized to 1.0 in all measured data. The mixing of the two solutions is not simply one-directional, i.e. from right fluorescein side to left DI side of the graphs in this experiment. The mixing region is simultaneously advancing in both directions from the interface due to the rotating effect and the transversal advection-diffusion effect, especially in the mixing of the two solutions [18,27,28]. This is observed by the lower fluorescence on the right half side of the graph after 1 cycle (red lines in Fig 3) in three operation modes, i.e. positive forward, positive reverse, and negative forward. However, it was not observed with negative reverse flow. We assume that relatively weaker mixing caused this. A full scale fluorescence intensity plot of the negative reverse setup is displayed in Fig 3E. The required number of cycles for complete mixing in positive forward, positive reverse, negative forward, and negative reverse is measured as two, two, four, and, five, respectively. Obviously, the positive SHM shows the highest mixing efficiency among tested operation modes. Interestingly, the positive reverse SHM shows mixing efficiency similar to the positive forward, even though it was lower after the first cycle. Considering the even lower Reynolds number in the positive geometry due to the smaller microchannel volume, the positive pattern has better mixing efficiency than the conventionally used negative SHM. However, further experimental and numerical investigations of the mixing efficiencies from different geometries, for example, different spacing and height of individual grooves can enable in-depth understanding of the correlation between surface structure and surrounding fluids. The complete graphs of the normalized fluorescence intensities are shown in the supplementary material (S2 Fig).

### Numerical simulations of the SHM

**Fig 4:**
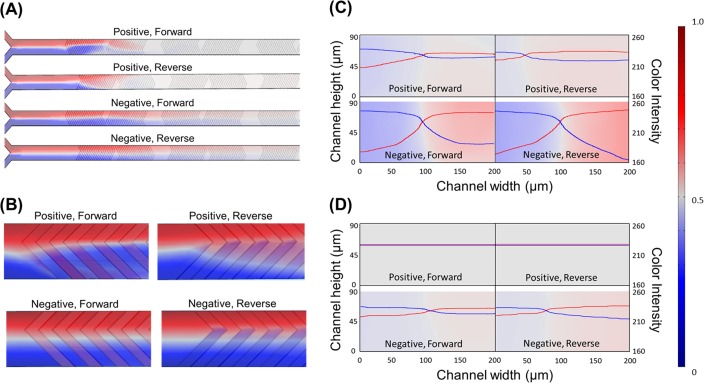
Numerical simulation results of the SHM devices. Red and blue color indicates fluorescein and DI, respectively. The white color code represents complete mixing. (A) Top view images obtained from the 3D simulation of the SHM up to 2.5 cycles (the width of the microchannels in the images are magnified 2 times for better observation) (B) Top view images of simulated flow at the entrance of the first cycle (C-D) Cross sectional images and color intensity of each fluid in the microchannel (C) After first cycle (D) After third cycle. The color legends present fluorescence dye (red) and DI (blue) concentrations.

The three-dimensional simulations successfully demonstrate the behaviors of the fluid obtained from the experiments, and the results are displayed in Fig 4. The cross sectional images of two aqueous solutions in the channels are simulated after each cycle of the SHM pattern. Fig 4A shows the mixing status of the two streams along the distance from inlet up to 2.5 cycles, which is 7550 μm. Before entering the first cycle, the two solutions are rarely mixed and form a laminar flow. This result is also clearly shown in Fig 4B, which is the magnified view at the entrance of the SHM structure. The simulation results well describe the behavior of fluids at the entrance of the positive and negative SHM structure obtained from the experiments as shown in Fig 2A–2D. Fig 4C shows the status of mixing right after the first cycle. The richer color indicates less mixing, and thus negative SHM structures have lower mixing efficiency than positive structures. This is also supported from the meniscus of two fluids clearly shown in negative structures. In addition, the color intensity plots displayed as red and blue lines present the concentration profiles of fluorescence (red) and DI (blue) across the microchannel. Again, the large gaps between red and blue lines in negative patterns indicate lower mixing efficiency compared with positive patterns. The negative reverse setup has the lowest mixing efficiency among tested structures, since the gaps between the red and blue lines are largest near the side walls. Interestingly, the simulation results show that the meniscus between the two fluids is closer to the DI side in the positive reverse structure. This is more clearly shown from the location of the crossing point of the red and blue lines. The fluorescence solution transferred toward the DI side up to around 50 μm of the microchannel wall, while that of the positive forward is around 100 μm. Considering the similar mixing efficiencies from both forward and reverse flow with positive SHM after the second cycle, the reverse pattern has an even higher directional transfer effect than the forward pattern. This result is expected to be a reason of the asymmetric fluorescence profile after the first cycle in the positive forward pattern, as shown in the red line of Fig 3A. This asymmetric profile is also visible in the fluorescence picture after the first cycle, i.e. captured image below Fig 2A.

The numerical simulation also shows that the mixing in the positive forward and reverse patterns are completed in three cycles, and the color scheme after the third cycle is shown in Fig 4D. After the third cycle, the color intensity plots of the two solutions are similar level flat lines in both the positive forward and reverse patterns, while that of the negative patterns indicates incomplete mixing. In the numerical simulations, the required number of cycles for complete mixing in positive forward, positive reverse, negative forward, and negative reverse is calculated as two, two, five, and, five, respectively. The number of cycles required for complete mixing was determined by comparing the color intensity profile using the ANOVA test with a 95% confidence level. Only the required number of cycles for the negative forward was different from the experimental results. The reason for this discrepancy is assumed to be the different approaches to analyzing the fluorescence dye concentration. In the experiment, the fluorescence dye concentration was observed and analyzed through the projected area obtained from top-view microscopic images. In this case, uneven mixture schemes of the liquids depending on the height in the microchannel are obscured in top-view images [24,25]. The woven boundary of the two fluids depending on the height in negative patterns clearly shows the possibility of this effect, as shown in Fig 4C. On the other hand, in numerical simulation, the fluorescence dye concentration can be analyzed via cross-sectional plane view images to clarify details of the liquid mixing inside the microchannel. The numerical simulation results confirmed that the SHM mixers with positive convex grooves improve the mixing efficiency as observed in the experiment. The cross-section analysis of each cycle by numerical simulation is presented in the supplementary material (S3 Fig).

**Fig 5:**
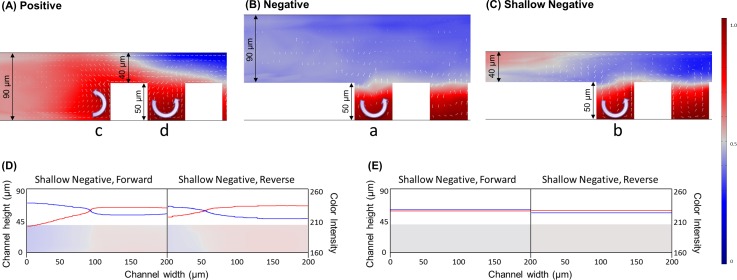
Numerical simulation of the fluid behavior at SHM entrances. (A) Positive forward (B) Negative forward (C) Shallow negative forward. Arrows indicate relative vorticity of the fluids regarding x and z axis of the channel. (D-E) Cross sectional images and color intensity of each fluid in the microchannel. (D) After first cycle of shallow negative. (E) After third cycle of shallow negative. The color legends present concentrations of fluorescence dye (red) and DI (blue).

Numerical simulations of the shallow device considering the number of cycles required for complete mixing was two and five for positive and negative forward setups, respectively; better mixing efficiency from positive patterns were confirmed by experiments and numerical simulations. However, the total volume of the positive pattern device, i.e. 0.47 μL, is smaller than negative pattern device, i.e. 0.61 μL, due to the volume occupied by SHM structure. Even the fluids are laminar with low Reynolds numbers of 0.229 and 0.097 for positive and negative patterns, respectively; this difference in volume can affect the mixing efficiency. The differences of the positive and negative pattern devices are described in Fig 5A and 5B. Since the heights of the channels in the branch area are the same for both the positive and negative patterns, the distance between cover to groove surface is 40 or 140 μm, respectively. Thus, a shallow device that has lowered height in the branch area and the same size negative grooves was designed and tested by numerical simulation. The dimensions of the shallow device are shown in Fig 5C. The ratio between total height (indicated a in Fig 5) and groove depth (indicated b in Fig 5) of the positive and negative patterns used in the experiments were 0.556 and 0.357, respectively. The ratio in the shallow device is calculated as 0.556, which is identical to the ratio of the positive pattern device. The total volume and Reynolds number is 0.33 μL and 0.123, respectively. Fig 5D and 5E indicate the statuses of mixing after the first and third cycles in shallow negative devices with forward and reverse flows. The height of the channel in the branch area is 40 μm, which is less than half of the positive and negative pattern used in the experiments. The mixing efficiency was improved in shallow devices with both forward and reverse flows compared with the thicker negative patterns. In the shallow negative patterns, the required number of cycles for complete mixing in forward and reverse flow was three and four, respectively. The ratio between channel height and groove depth is the same for the positive pattern and the shallow negative pattern device, as mentioned earlier; however, the positive pattern still shows better mixing efficiency. This can be explained if the positive convex shape at the entrance of SHM structure enhances mixing. Fig 5A–5C present cross-sectional mixing status and relative vorticities of the two streams at the entrance of positive forward, negative forward, and shallow negative forward pattern channels, respectively. The cross-section is obtained from the middle of the channel. For the vorticity arrow, the magnitude to y-axis (direction to the side wall of the channel) is neglected to investigate two-dimensional mobility. In the case of the negative (Fig 5B) and the shallow negative pattern (Fig 5C), fluids formed a vortex after they were introduced to the groove of the concave pattern (Fig 5B-a and 5C-b); However, in the case of a positive pattern, the flow of the fluids started to form a larger vortex through impinging directly on the convex patterns as indicated in Fig 5A-c, as well as small vortex in the area between two positive grooves (Fig 5A-d). Entire volumes of the positive, negative, and shallow negative patterns used in the study are 0.47, 0.61, and 0.33 μL, respectively. Thus, the positive pattern can mix larger amounts of the liquids more efficiently compared with the negative pattern. These results support that the SHM mixers with positive convex grooves can have higher mixing efficiency than conventionally used negative concave grooves.

**Fig 6:**
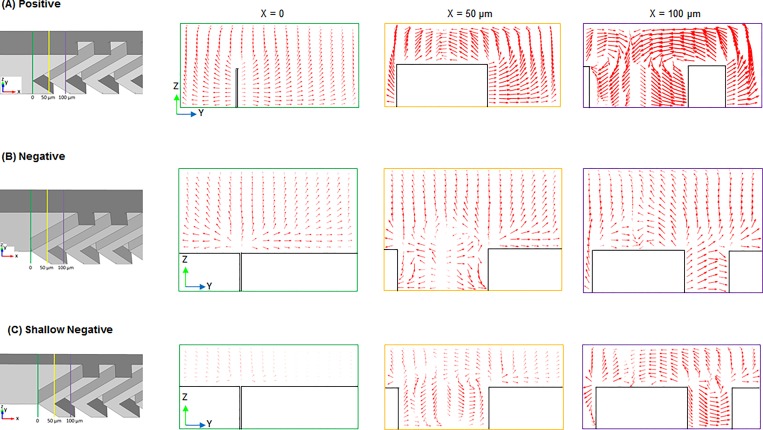
Cross-sectional velocity vector plots at SHM entrances. (A) Positive forward (B) Negative forward (C) Shallow negative forward. Arrows indicate relative velocity of the fluids regarding y and z axis of the channel. Fluidic velocity in the positive pattern is seven and two times larger than the negative and shallow negative pattern, respectively, when the size of the arrow in simulation is the same due to scale factor difference.

The velocity vector plots are shown in Fig 6, on the yz-plane which is perpendicular to the main flow direction (x-plane) from the inlets to the outlets of the positive forward, negative forward, and shallow negative forward pattern channels. For the velocity vector arrows, the magnitude to x-axis (direction to the outlet of the channel) is neglected to investigate two-dimensional mobility in y and z directions. The cross-sectional images of fluid behaviors at the entrances of first SHM pattern are explained by vector plots in Fig 6 depending on the distance from edge of the first groove, from 0 to 100 μm. The vacant areas in the vector plots indicated the cross-section of the convex or concave parts of the grooves on the positive or negative pattern. As indicated by the size of arrows, the velocity of the stream is higher in the middle of groove (100 μm) in all three cases. The positive pattern has largest vortices comparing with other two types. In this simulation, the scale factor of the vector arrow for the negative pattern and shallow negative pattern was set seven and two times larger than that of positive pattern for better visibility of the arrows, respectively. Thus, if the size of the arrows are the same in all three patterns, fluidic velocity in the positive pattern is seven times greater than the negative pattern, and twice greater than in negative shallow pattern. These comparisons clearly support that the fluids form a larger vortex in positive pattern than those of the negative and the shallow negative patterns.

### Mixing with SHM in viscous fluids

**Fig 7:**
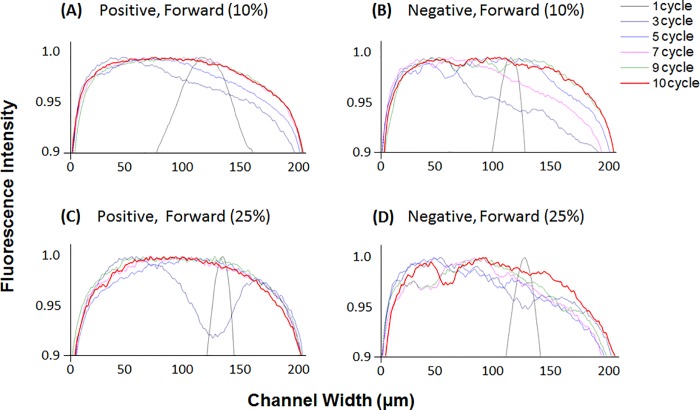
Normalized fluorescence intensity across the microchannel in viscous fluid test. after each cycle from 1 to 10. (A) 10% Positive forward, (B) 10% Negative forward, (C) 25% Positive forward, (D) 25% Negative forward.

The mixing efficiency of the SHM devices was tested with viscous solutions for further applications in variety of experiments. DI water–glycerol solutions (10% and 25% (w/v), where 0% glycerol is only DI water) were used to study the performance of the SHM on viscous fluids, and the results are displayed in Fig 7. Both viscous glycerol fluids were not completely mixed even passing through all of the 10 cycles patterned in both of positive and negative SHM; however, positive pattern shows higher mixing efficiency than negative. After the end of all of the 10 cycles, the fluorescence intensity of the negative SHM is still unevenly distributed across the channel different from relatively evenly mixed the positive SHM (Fig 7A–7D). It is clearly shown after tenth cycle (red line in Fig 7).These results support better mixing effect of positive pattern than negative SHM even with the viscous fluids. The graphs of all 10 cycles are shown in the supplementary material (S4 Fig).

However, further investigation on the three-dimensional behavior of the fluid in SHM is required. Confocal imaging method is appropriate for this purpose as performed in previous analysis using confocal imaging [10,30]. The discrepancy between simulation and experimental results may occur due to a non-uniform mixing at the edges of the channel, or a multi-layered mixture after the end of each cycle in the actual experiments, unlike ideal mixing in the simulation. It is expected that confocal imaging method will confirm the better mixing efficiency in positive SHM pattern with more accurate analysis of fluid behavior in SHM.

## Conclusions

In this study, the effect of SHM geometries on the mixing of two liquids was investigated by experiments as well as numerical studies. Specifically, the novel positive pattern shows higher mixing efficiency than the conventionally used negative pattern. The direction of the flow also affects the mixing efficiency in the SHM. In general, forward flow shows better mixing efficiency than reverse flow with a negative pattern. The conventional negative pattern requires four cycles with forward flow for complete mixing, and thus has roughly two times lower mixing efficiency than positive forward flow. The numerical simulation confirmed that the positive SHM devices perform better mixing efficiency than even shallow negative SHM devices. The developed passive SHM with a positive pattern can be easily integrated in a microfluidic device to enhance the mixing efficiency for various biological and chemical reactions. This finding can help to interpret the phenomena of positive and negative patterns existing in nature, depending on the function of those patterns.

## Supporting Information

S1 Fig Picture of the used device. (PDF)

S2 Fig Normalized fluorescence intensity across the microchannel after each cycle. (PDF)

S3 Fig Simulated fluorescence intensity across the microchannel after each cycle. (PDF)

S4 Fig Normalized fluorescence intensity across the microchannel in viscous fluid test. (PDF)
