# Supplementary material for: Convex Grooves in Staggered Herringbone Mixer Improve Mixing Efficiency of Laminar Flow in Microchannel
Source: PLoS One. 2016 Nov 4;11(11):e0166068. doi: 10.1371/journal.pone.0166068 (PMC5096722; doi:10.1371/journal.pone.0166068)
Supplement: S1 Fig — (PDF) [file pone.0166068.s001.pdf]

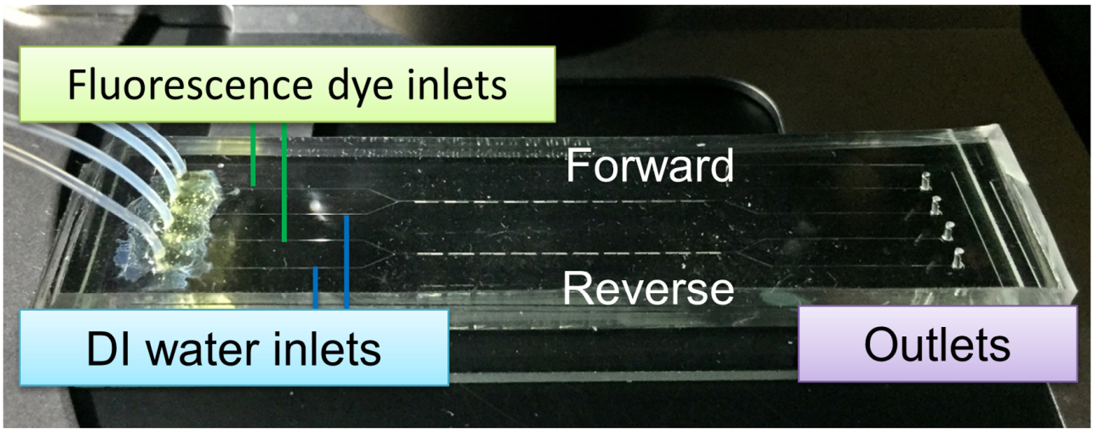

S1 Fig. Picture of the used device. Intermittent bright lines in main straight channel indicate each cycle of SHM.

Since convex and concave patterns have different total heights, two casting molds were fabricated. Each mold has forward and reverse patterns to minimize height variation during photolithography. Another microchip, which has no patterns inside, was separately fabricated. Each micro-mixer chips consisted 10 cycles of SHM patterns. The devices were fabricated by PDMS (polydimethylsiloxane) replica casting using mold made by photolithography process.
