# Supplementary material for: Convex Grooves in Staggered Herringbone Mixer Improve Mixing Efficiency of Laminar Flow in Microchannel
Source: PLoS One. 2016 Nov 4;11(11):e0166068. doi: 10.1371/journal.pone.0166068 (PMC5096722; doi:10.1371/journal.pone.0166068)
Supplement: S2 Fig — (PDF) [file pone.0166068.s002.pdf]

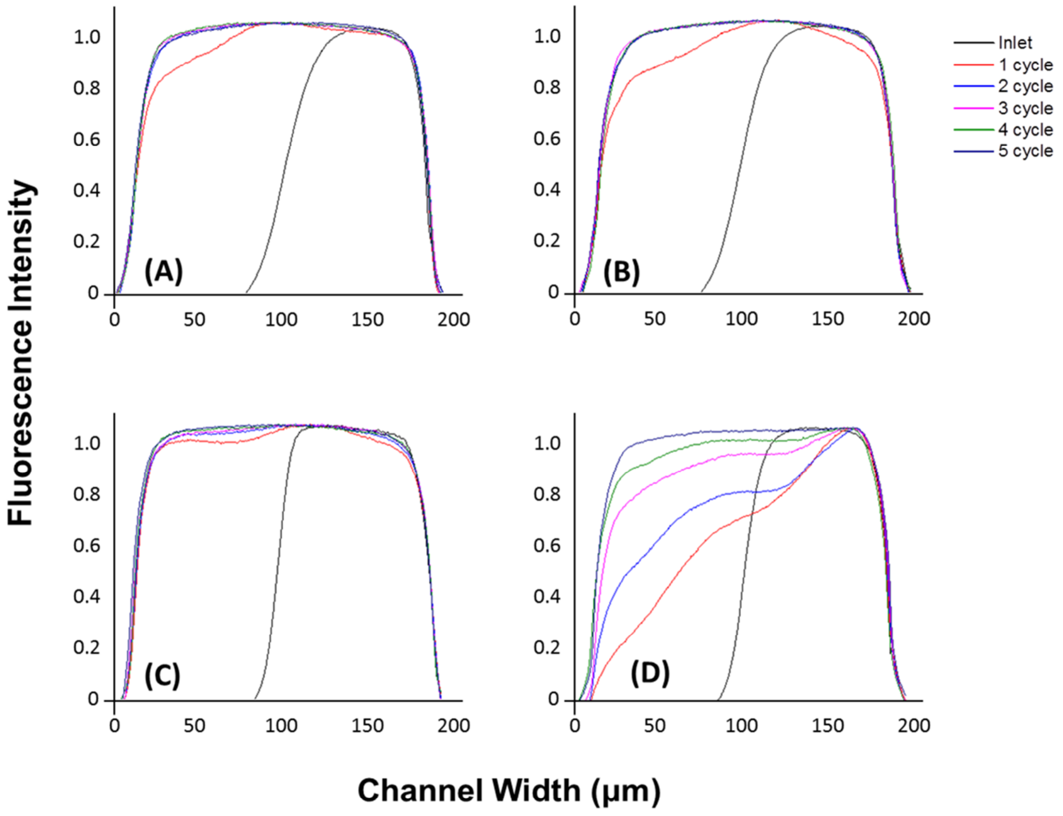

S2 Fig. Normalized fluorescence intensity across the microchannel after each cycle from 1 to 5. (A) Positive forward (B) Positive reverse (C) Negative forward (D) Negative reverse

The intensity of the fluorescence across the microchannels was scanned after each cycle from 1 to 5 and is displayed in S2 Fig. The obtained intensity from the scanning of the captured fluorescence image was normalized in each analysis, by dividing all the obtained brightness by the maximum brightness of the fluorescence in each set of data. Thus, the maximum brightness is set to 1.0 in all measured data.
