# Supplementary material for: Convex Grooves in Staggered Herringbone Mixer Improve Mixing Efficiency of Laminar Flow in Microchannel
Source: PLoS One. 2016 Nov 4;11(11):e0166068. doi: 10.1371/journal.pone.0166068 (PMC5096722; doi:10.1371/journal.pone.0166068)
Supplement: S3 Fig — (PDF) [file pone.0166068.s003.pdf]

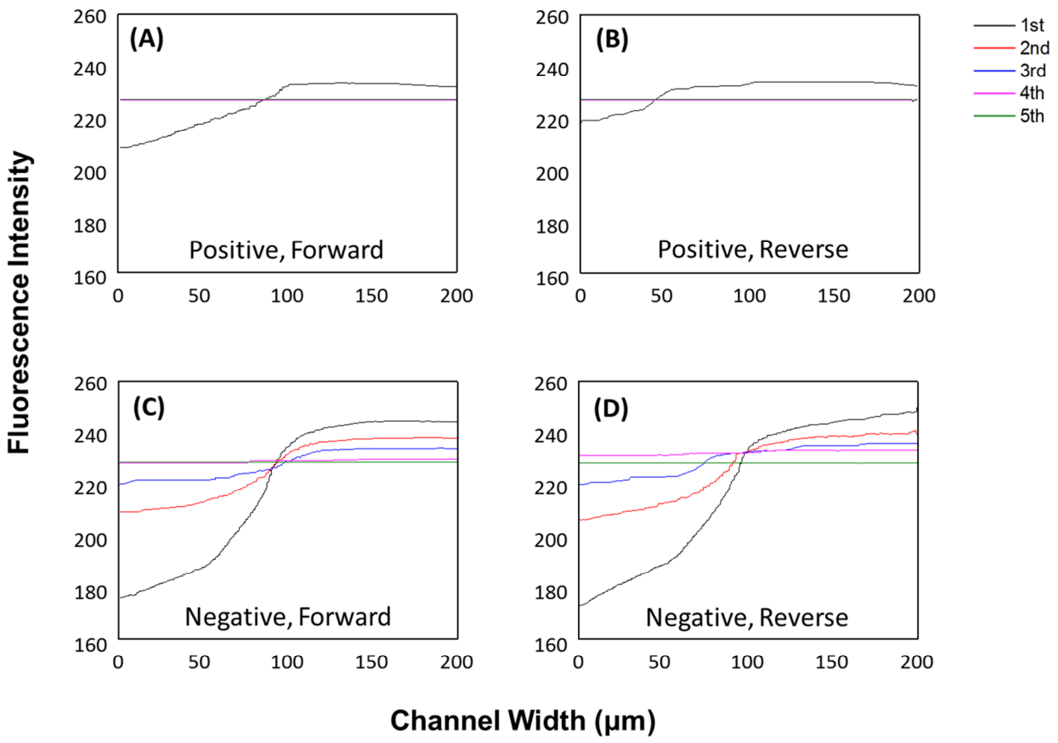

S3 Fig. Simulated fluorescence intensity across the microchannel after each cycle. (A) Positive forward (B) Positive reverse (C) Negative forward (D) Negative reverse

The concentrations of the fluorescence dye across the microchannels were analyzed after each cycle from 1 to 5 and displayed in S3 Fig. The numerically simulated image after each cycle was examined by cross-sectional images as fluorescence dye concentration. The maximum concentration is arbitrarily unit of fluorescence intensity set to maximum of 255 in all measured data.
