# Supplementary material for: Convex Grooves in Staggered Herringbone Mixer Improve Mixing Efficiency of Laminar Flow in Microchannel
Source: PLoS One. 2016 Nov 4;11(11):e0166068. doi: 10.1371/journal.pone.0166068 (PMC5096722; doi:10.1371/journal.pone.0166068)
Supplement: S4 Fig — (PDF) [file pone.0166068.s004.pdf]

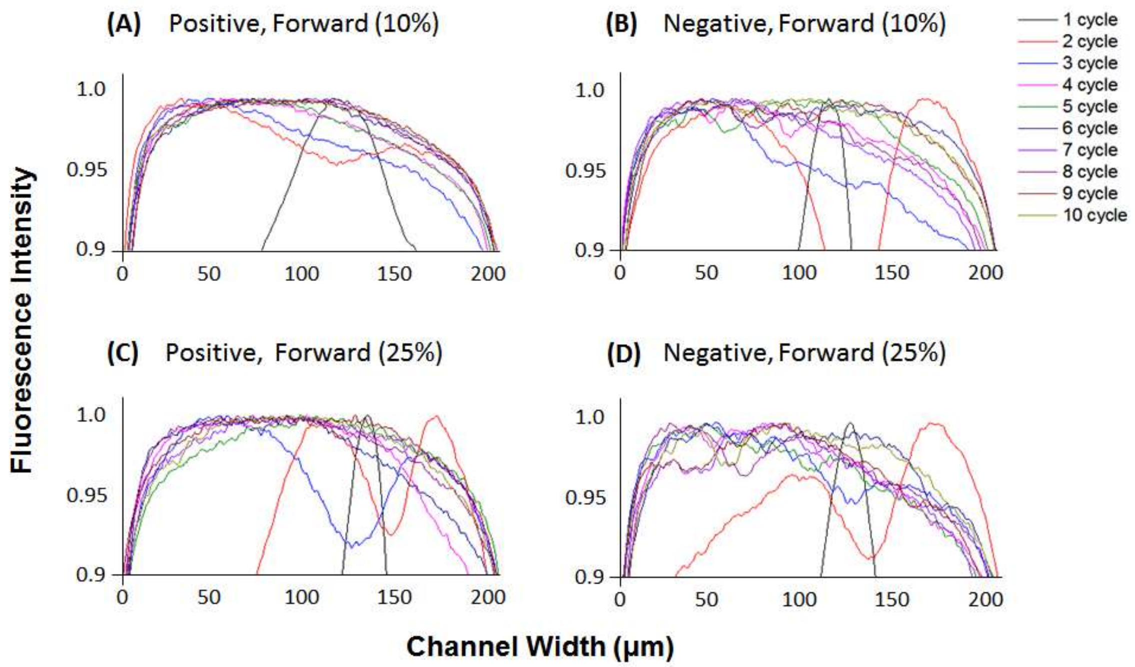

S4 Fig. Normalized fluorescence intensity across the microchannel in viscous fluid test. After each cycle from 1 to 10. (A) 10% Positive forward, (B) 10% Negative forward, (C) 25% Positive forward, (D) 25% Negative forward.

The mixing efficiency of the SHM devices was tested with viscous solutions. The concentrations of the fluorescence dye across the microchannels were analyzed after each cycle from 1 to 10 and displayed in S4 Fig.
